# Solutions for medical databases optimal exploitation

**Published:** 2014-03-25

**Authors:** I Branescu, VL Purcarea, R Dobrescu

**Affiliations:** * Faculty of Automatic Control and Computers, Polytechnic University of Bucharest; ** Department of Healthcare Marketing, Technology and Medical Devices, Medical Informatics and Biostatistics, “Carol Davila" University of Medicine and Pharmacy, Bucharest

**Keywords:** OLAP technique, medical database, pre-aggregation, EPR, data warehousing

## Abstract

The paper discusses the methods to apply OLAP techniques for multidimensional databases that leverage the existing, performance-enhancing technique, known as practical pre-aggregation, by making this technique relevant to a much wider range of medical applications, as a logistic support to the data warehousing techniques. The transformations have practically low computational complexity and they may be implemented using standard relational database technology. The paper also describes how to integrate the transformed hierarchies in current OLAP systems, transparently to the user and proposes a flexible, “multimodel" federated system for extending OLAP querying to external object databases.

## Introduction

The integration of biomedical information has become an essential task for health care professionals. Current progress in the domain of Information Technology allows for huge data storages and powerful computational possibilities to be affordable; thus, they have been quite common. Researchers are gradually becoming aware of the importance of keeping together diverse data pertaining to a specific medical entity and successful attempts to create and maintain such databases are becoming known to the scientific community [**1**]. Data models specified by standards are often included in databases, without taking into account inherent limitations posed by the procedure of acquiring original data. It is therefore quite often that inference is affected by errors that propagate throughout the entire process, from data acquisition through processing and analysis. While data models are adequate for their initial usage, they are inflexible to the requirements posed by new analysis procedures that become available as data are massively aggregated from diverse origins. Often, they include data reduction steps, narrowing the scope of future analyses. Having the data in their raw form, however, would offer the opportunity of reanalyzing the data as new, unpredicted at the time of acquisition, hypotheses are put under test. Integration is therefore much more than a plain collection of digital biomedical data [**2**]. Homogenization of data description and storage, followed by normalization across the various experimental conditions would be a prerequisite for facilitating procedures of knowledge extraction [**3**].

 Being an application area where complex multidimensional data is common within medical informatics, the beneficiaries may benefit significantly from the functionality offered by two important IT achievements: data warehousing (DW) and Online Analytical Processing (OLAP). However, the special nature of clinical applications poses different and new requirements to data warehousing technologies, over those posed by conventional data warehouse applications. These include the need for complex-data modeling features, advanced temporal support, advanced classification structures, continuously valued data, dimensionally reduced data, and the integration of complex data. OLAP systems typically employ multidimensional data models to structure their data. The modeling requirements for multidimensional medical data models are derived from a realistic assessment of complex data found in real-world applications. In particular, we discuss a model that reuses the common multidimensional concepts of dimension hierarchies and granularities to capture imprecise data. The presented data model and query evaluation techniques can be implemented using relational database technology. The approach is also capable of exploiting multidimensional query processing techniques like pre-aggregation. This yields a practical solution with low computational overhead. Pre-aggregation, the prior materialization of aggregate queries for later use is an essential technique for ensuring adequate response time during data analysis. Full pre-aggregation, where all combinations of aggregates are materialized, is infeasible. Instead, modern OLAP systems adopt the practical pre-aggregation approach of materializing only select combinations of aggregates and then re-use these for efficiently computing other aggregates. However, this re-use of aggregates is contingent on the dimension hierarchies and the relationships between facts and dimensions satisfying stringent constraints. Moreover specifically algorithms are given that transform “irregular" dimension hierarchies and fact-dimension relationships, which often occur in real-world OLAP applications, into well-behaved structures that, when used by existing OLAP systems, enable practical pre-aggregation. The algorithms have low computational complexity and may be applied incrementally to reduce the cost of updating OLAP structures. The transformations can be made transparently to the user.

**Medical information structures**

*Medical data*

 Information is collected using dissimilar collection and measurement methods (such as physical examinations, lab tests, radiology tests, patient interviews). Measurement equipment has different accuracy ranges and doctors have different education and experience levels. Values can be difficult to compare between patients and measurement opportunities. Information coming from patient interviews is subjective and its usability largely depends on the ability of physicians to ask correct questions, interpret the answers and later codify the information correctly. Routines for entering patient data in EPRs might also affect the coverage and consistency of the data sources. This problem can only be addressed by better software that permits effective and suitable structured data entry and stricter quality control.

*Molecular biology data*

 In bioinformatics, technological breakthroughs are producing unprecedented massifs of data. Genomics sciences are experiencing a paradigm change where comparison of whole genomes is a real possibility. The medical applications are also important since these new technologies enable cost-effective personalized medicine as the cost of genome sequencing continues to decrease. Several breakthroughs have resulted in very cost-effective Next-Generation Sequencing (NGS) technologies [**4**]. Selection of NGS techniques is influenced by the intended goal, for example genome assembly, transcriptomic analysis, single nucleotide polymorphism (SNP) or copy number variations (CNV). In all those cases, the amount of data to process is considerable. Cost of data production is also decreasing. Consequently, genome sequencing of patients, as a base for personalized medicine, will likely become economically viable and common in the near future, and many genome-disease associations will be previously known for such sequencing to influence determination of diagnosis or therapy. 

 Other areas in bioinformatics are also experiencing dramatically growing data sizes as a result of technology advances, for example in DNA microarrays [**5**]. Taking into account such data production, it is also essential to consider data quality aspects. Unfortunately, data curation lags behind the rapid generation of data. The field of biocuration aims to organize, represent and make available biological data to researchers. Tasks such as data representation, gene annotation in large databases and tagging research papers with concepts from controlled vocabularies are considered crucial for future research. 

*Sensitive patient data*

 Patient data is sensitive information and is, therefore, protected by privacy laws. For example, there is a risk that medical insurance companies might deny coverage based on the medical history of patients. With the increased citizen mobility in the European Union, interchange of medical history of patients becomes a necessity. However, legislation concerning handling of medical data make it difficult, or even impossible, to attain necessary permissions to use data across country borders. Many hospital information systems have traditionally been constructed as “islands of data" [**6**]. To some extent, this is a direct effect of health care organizations policies to build a secure “wall" around their information systems. Sharing of patient data with other caregivers outside the organization is thereby limited although such sharing can be motivated both ethically and medically. There are similar ethical concerns for molecular biology data since genetic data from patients can be used to identify a person. Hence, any research involving genetic data must also consider ethical and legal issues. However, it is not always necessary to know the identity of the person from which data comes from. Various procedures of anonymization then become possible. Truly anonymous data, which is impossible for anyone to associate to persons and pseudonymised data where information that can be used for personal identification has been replaced with a label. The association between labels and personal information is only possible if the researcher has access to a mapping file. If the researcher does not have such access, the genetic data can be considered anonymised from the point of view of the researcher. In [**7**], the architecture for data protection in the European Union co-founded project Advancing Clinico Genomic Trials (ACGT) is described. The high-level objective of the ACGT project is the development of methods and software systems that can improve the understanding of cancer research data through integration and analysis of biomedical information. This is accomplished by the development of an integrated and Grid compatible software platform, which can support post-genomic, multi-centre clinical trials and allows the pseudonymisation of sensitive data and the establishment of an authority for data protection, which enters into legally binding agreements with project participants to ensure data protection. 

**Integration of heterogeneous medical data sources**

 One early effort to standardize patient record structures is the Patient Record Architecture (PRA) document architecture which suggested to use the information model in Health Level 7 (HL7) [**8**] as a reference to exchange patient data records. Despite such efforts, the multitude of sources and structures of data related to patient care remain great challenges for data integration and communication. However, integration and communication are fundamental to enable secondary usages of patient data, such as discovery of clinically interesting patterns in large data sets or integration with biomolecular data (enabling personalized medicine). This is the foundation for the development of clinical guidelines and Clinical Decision Support Systems (CDSS). There are, of course, many approaches to data integration but here we only highlight two approaches that have been applied previously in medical informatics: data warehousing and mediation-based approaches.

*Terminology systems in medicine*

 Codification of medical data is important for efficient information interchange. Such codification assigns terms from terminology systems to patient data. A terminology system assigns terms to concepts or objects within a certain domain. Patient data can thereby be understood with considerably less ambiguity, assuming that the terminology system accurately represents the domain both in scope and in granularity. A multitude of terminology standards in medicine are available, see [**9**] for overviews. Relations between concepts in a terminology system can imply additional information. Two types of hierarchical relations can be differentiated. Generic relations arrange terms in the sense that the species possesses all features of the genus with at least one other feature added, for example, “Hepatitis B" (species) is a “viral hepatitis" (genus). Another kind of hierarchical relation is the partitive relation, which is the relation between the whole and the parts, for example “heart" (part) and “vascular system" (whole). There are also non-hierarchical relations such as relations between concepts based on time, space and cause. Especially time relations are important in medicine since they enable analysis of time-series and thereby trends.

 The codification process is affected by the choice of terminology systems. Selection of standards is consequently important. Several aspects are important for terminology systems [**10**]:

 • Domain completeness

 • Non-overlapping classes (clear criteria for class boundaries)

 • Adequacy for its purpose

 • Homogeneous ordering (one principle per level)

 • Usage guidelines

 • Appropriate level of detail

 Several types of terminology systems can be differentiated. A thesaurus is a terminology with index and possibly synonyms and preferred terms. A classification is a terminology system in which concepts are related generically. A vocabulary is a terminology together with definitions of terms. A nomenclature is a terminology with rules to compose new concepts in a certain domain. A coding system is a terminology with a coding scheme. An ontology is a set of concepts together with their attributes and relations to other concepts.

 Medical data warehousing

 One approach to data integration is collecting and storing data in a data warehouse (DW). A DW is a “subject-oriented, integrated, time-variant, non-volatile collection of data in support of management decisions" [**11**]. DWs are suitable for data mining since data in a DW is non-volatile and analysis is therefore repeatable. Data is loaded, transformed, cleaned and regularly updated from the data sources to the data warehouse. Potential conflicts and inconsistencies between the data sources must also be resolved. Differences in naming, structures, semantics and implied knowledge in the data sources could complicate integration. Online analytical processing (OLAP) tools are used for multidimensional analysis in DWs. OLAP permits analysts to successively focus on lower levels of detail.

 DWs are also better suited than EPRs for analysis of patient data since the database structures of EPRs are designed for efficient access to data on a patient specific basis. This causes efficiency problems when queries that are more general are needed.

 Typically, normalized data are stored in tables to save both space and ensure consistency.

 Since DWs are intended for data mining, they can also achieve better efficiency by re-organizing data and reducing the necessary table conjoining during query processing. Depending on the level of structure and quality of patient data coming from data sources, data imported into the DW might need some “scrubbing" (for example by codifying existing data). Data in a DW are collected from multiple data sources and transformed to use common data formats and terminology. This is a complicated task in medical DWs, as it involves collection and integration of data from various health care information systems (for example EPRs, nation-wide clinical registers and administrative databases containing patient billing information). The medical history of a patient can be of great importance in data analysis. If such patient data is stored in external information systems (for example, when a patient has moved and changed hospital), it becomes necessary to identify the patient across data sources (using social security numbers or other strategies) and ensure that information is codified using the same terminology system.

 In [**12**], a methodology is proposed for establishing DWs in medicine. A DW to support long-term care of elderly patients is used to exemplify the methodology. Decisions have to be made regarding intervals of data updates and protocols. It is important to establish trust among the owners of the data sources that patient data privacy is a high priority. Data is anonymised before being exported to the DW but each data source owner could, if needed, use a mapping between social security numbers of the patients and the internal identifiers in the DW system to identify patients. Reported problems were related to the quality of the data, which resulted in the development of complex filtering procedures (in some cases even manual inspection was needed). The use and development of ontologies could be seen both as a requirement and as an important result from data integration since it motivates stakeholders (data source owners) to use common terminology for data. Differences in terminology system usages were resolved during data transformation and pre-processing. The significance of DWs for evidence-based medicine is discussed in [**13**]. Generation of logic for guidelines is suggested to be based directly on data mining of clinical data. Several case studies are used as a motivation for important tasks that can be supported by DWs concerning evidence-based medicine. Rules in guidelines can be continuously refined as additional data is included in the data warehouse. DWs can also support the development of clinical pathways (i.e. human-oriented processes) through optimization of resources in a clinical environment based on past patient data, for example average hospital stay for recovery after operations. The main usage of the data repository is to generate administrative reports although some examples of medical queries are given.

 Different Biomedical Informatics Grid offer the software infrastructure to facilitate biomedical research by extending existing Grid computing technologies and infrastructure with programmatic support for integrating structure and semantic aspects of biomedical data. Grid services can be discovered based on complex queries including input/output data semantics and other characteristics (statistics related to availability of services etc.). Service compositions can be constructed and enacted as workflows. While there are different levels of integration in a grid, ideally services and data should be made interoperable by using common vocabularies to facilitate data integration, including annotation of data elements (meaning), value domains (which values an element can have), relations to other concepts and syntactic integration by sharing XML schemas. Services should also be annotated with metadata such as input and output data types and under which authority (organization) the service is provided. Extraction, transformation and loading (ETL) of data from heterogeneous data sources is facilitated with semantic information. The ETL process was based on ontologies that were generated using descriptions of the data sources in UML. For example, in caGrisd [**14**] the ontologies were loaded into a semantic triple store. Data was extracted from the two data sources and linked with a custom inference rule. The data was then loaded into a “semantic" data warehouse. Their approach is demonstrated by querying data from the data warehouse and performing a Principal Components Analysis. The discussed solution shows the utility of using ontologies to improve data integration.

*Mediator-based methods*

 The establishment of data warehouses is not always an optimal solution. For example, the enormous data sizes being produced from NGS projects are not easily transferred. Accordingly, rather than collecting and storing data in a centralized repository, an alternative approach is to leave the data at the original data sources and instead dynamically combine data through queries resolved by a mediator. A mediator-based system in data integration performs dynamic translation of user queries between the schema of a mediator component and schemas of data sources. Data is not re-organized and expressed in common terminology system. Instead, mappings between a common mediator schema and data source schemas are established. Early bioinformatics systems for data integration consisted of federated databases where databases with different schemas can be queried as if they have a common schema. Mediator-based solutions can be seen as loosely coupled versions of federated databases [**15**].

In Global-As-View (GAV), a global schema is established with direct mappings to the local schemas. Adding new data sources requires a modification of the global schema and the mappings. The Local-As-View (LAV) approach is scalable and easier to maintain than GAV since the global schema is created independently of the local schemas. The schemas of new data sources are described in terms of the global schema (which thereby changes less frequently). LAV usually suffers from low query performance for very complex queries compared to GAV.

 The ACGT project [**16**] highlights the complexity of integrating patient and genomics data from multiple centers and even countries. Data integration is mediator-based where a master ontology describes the domain concepts. Patient data is anonymised and stored in (potentially) heterogeneous databases. Data-sources are represented by a source description consisting of a local ontology with mappings to the local data source schema together with references to concepts in the master ontology (a LAV approach). Additionally, source descriptions contain metadata specifying query capabilities and security information. Queries are specified in terms of the master ontology concepts. Users (including ACGT services) do not need to be aware of data source schemas or access methods in the participating centers. Queries are sent to a mediator component that translates the queries to use terms from the local ontology of the data source. The translated queries are sent to wrappers that use the source description to form a query expressed in the local database schema. The result from the local database query is translated to use concepts of the local ontology and sent to the mediator that translates the data to use terms from the master ontology and integrates results from potentially several sources. Results from queries or data mining software are shared in a common storage system.

*Issues to be solved*

 There are still many factors to be considered when designing any system that deals with patient data. The usage of consistent standards must to be a priority in order to promote secondary uses of patient data. Because of semantic problems when dealing with codes in different terminologies, heterogeneous data from many sources are difficult to correctly integrate and interpret. Identification of similar terms (aliases) among various terminology systems is error-prone since the systems can differ in structural organization, semantics and in granularity of concepts. By adding additional information, such as temporal information (when was the value entered in the system), the source of the information (measuring equipment model, doctor, nurse, CDSS advice etc.), patient history (previous examinations and diagnoses), what ranges of values are normal (or possible), we can describe the background of the data (i.e. metadata). Even references to the actual workflow at the source clinic can be specified, allowing data analysts to determine if data are trustworthy.

 Another important aspect is the coverage of medical terms. It is not sure that a single standard can be used for all fields in medicine. This is, in part, the motivation for the multitude of standards in medicine. In some cases, existing standards have been adapted for use in specialty fields. Secondary uses, such as statistics and knowledge extraction, make it difficult to predict who will use the data.

 Mappings of medical terminology systems are likely to be necessary when integration of heterogeneous data sources is pursued. The Unified Medical Language System (UMLS) attempts to unify medical terminologies by using a metathesaurus. It enables code translations between the terminology systems (nearly one hundred, counting national variants) which is an unavoidable requirement when faced with heterogeneous patient data sources. The metathesaurus itself already provides relations between clinical concepts. This is further specified in the semantic network of UMLS. Biomedical terms are specified in a specialist lexicon. Being able to communicate data between systems using different terminologies is not only recognized as an important feature by UMLS but also by more traditional terminology systems such as Read codes (also called the Read Clinical Classification). Additionally, genetic information and, in particular, patient data are protected by data-privacy legislation. Transferring such data is complex from a legal point of view, in particular over country borders.

 Most of these problems can be addressed by using an appropriate medical data model. 

**Development of a multidimensional medical data model (MMDM)**

*Requirements for MMDMs*

 The following requirements are considered mandatory for the elaboration of MMDM (see also [**17**] for details):

 1. Explicit hierarchies in dimensions: The simple cube models do not capture the hierarchies in the dimensions explicitly. Some models provide partial support by the grouping relation and dimension merging function, but do not capture the complete hierarchy together with the cube. 

 2. Symmetric treatment of dimensions and measures: Most of the models distinguish sharply between measures and dimensions. An attribute designated as a measure cannot be used as a dimensional attribute and vice versa. This restricts the flexibility of the cube designs.

 3. Multiple hierarchies in each dimension: Some models require that the schema of dimension hierarchies is tree-structured. To support multiple hierarchies, a more general lattice structure is required. 

 4. Support for aggregation semantics: Most of the models support aggregation semantics partially, by implicitly requiring the dimension hierarchies to be strict, onto, and covering, i.e., the hierarchies should be balanced trees. This is one of the conditions of summarizability and means that data will not be double-counted. 

 5. Non-strict hierarchies: Most of the models implicitly or explicitly require that hierarchies be strict. It is recommended that a model investigates the possible problems with allowing non-strict hierarchies and advises against using this feature.

 6. Many-to-many relationships between facts and dimensions: The models should allow many-to-many relationships between facts and their associated dimensions, such as the relationship between patients and diagnoses.

 7. Handling change and time 

 8. Handling different levels of granularity

 9. Handling imprecision: The concept of incomplete data cube, the most utilized MDM until now, provides partial support for imprecision in the data, as this can be handled using varying granularities. 

 Definition of the MMDM (after [**17**])

 An n-dimensional fact schema is a two-tuple S = (F;D), where F is a fact type and D = {Ti; i = 1; … n} is its corresponding dimension types.

 For example in a typical situation of editing a Patient File, we have Patient as the fact type, and Diagnosis, Residence, Age, Date of Birth, Name, as the dimension types. The intuition is that everything that characterizes the fact type is considered to be dimensional, even attributes that would be considered as measures in other models.

 A dimension type T is a four-tuple (C;PO;TE;BE), where C = {Cj; j = 1; … k} are the category types of T, PO is a partial order on the Cj’s, with TE and BE being the top and bottom element of the ordering, respectively. Thus, the category types form a lattice. The intuition is that one category type is “greater than" another category type if members of the former’s extension logically contain members of the latter’s extension, i.e., they have a larger element size. The top element of the ordering corresponds to the largest possible element size, that is, there is only one element in its extension, logically containing all other elements. We say that Cj is a category type of T. We assume a function that gives the set of immediate predecessors of a category type Cj. 

**Fig. 1:**
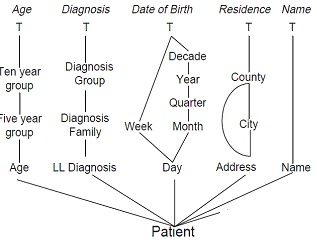
Graphical schema of a “Patient" model

 Low-level diagnoses are contained in diagnosis families, which are contained in diagnosis groups. Thus, the Diagnosis dimension type has the following order on its category types: BEDiagnosis = Low-level Diagnosis < Diagnosis Familiy < Diagnosis Group < TEDiagnosis. We have that pred(Low-level Diagnosis) = {Diagnosis Family}. Other examples of category types are Age and Ten-year Age Group from the Age dimension type, and DOB and Year from the DOB dimension type. Fig. 1 illustrates such dimension types.

 A category C has one or more representations. A representation Rep is a bijective function Rep: Dom(C) DomRep, i.e., a value of a representation uniquely identifies a single value of a category and vice versa. 

 A multidimensional object (MO) is a four-tuple M = (S;F;D;R), where S ) is the fact schema, F is a set of facts f where Type (f)=F, D = {Di; i = 1,…, n} is a set of dimensions where Type (Di)=Ti and R = {Ri; i = 1,…, n} is a set of fact-dimension relations. 

 A collection of multidimensional objects, possibly with shared subdimensions, is called a multidimensional object family.

 To summarize the essence of the model, the facts are objects with separate identity. Thus, we can test facts for equality, but we do not assume an ordering on the facts. The combination of the dimension values that characterize the facts in an MO do not constitute a specific key for the MO, in the sense that several facts may be characterized by the same combination of dimension values. But the facts of an MO is a set, so we do not have duplicate facts in an MO.

** New challenges in clinical data storage**

 Recent trends in medical information integration and exchange have focused on the use of new data warehousing solutions. Some of them are outlined in the following. 

 Utilizing EPR Features. One very important issue is to utilize the features of the EPR optimally for building a clinical data warehouse. All versions of data in the EPR are stored along with their times of update. The EPR is supposed to be the only (or at least the primary) tool that the clinical user is using in the daily work, so there is a great need to have access to all data, also lab results, etc., through the EPR. Thus, integration of operational data is already achieved in the EPR, making the integration process in the DW very easy in comparison to conventional data warehousing. 

 Complex Data Modeling Features. One of the most prominent demands is a data model that includes more complex modeling constructs than typical multidimensional models, while not losing their obvious strengths in the area of decision support, i.e., we should not return to the full generality of the ER model. 

 Advanced Temporal Support. One very important property of clinical data is the importance of temporal aspects. The same test can be made hundreds of times, so it is important to know both when the data is considered to be valid in the real world, and when it is stored and changed in the database. These temporal aspects of the data, known as valid time and transaction time, must both be supported to provide bitemporal support. This support is for instance needed in order to couple different facts (e.g., smoking and weight), thereby computing samples of measurement values at specific intervals or points in time. These samples are used to observe temporal trends in the evolution of one type of data values or in the relation between different types. In order to conduct these and other types of time studies, it is necessary to have available a strong support for time-series data, including a rich set of temporal analysis tools. 

 Advanced Classification Structures. A data type of extreme importance in the clinical sector is classified data. One example is a diagnosis, which at the lowest level is a very precise indication of one specific medical condition. Diagnoses are then grouped repeatedly into larger, more general classes. A diagnosis is a good example of a typical Online Analytic Processing (OLAP) dimension, as it characterizes the condition of the patient, it is attached to; but unlike the typical dimension, the diagnosis hierarchy is non-strict. The user of the DW should be able to work with the data and get the correct results, without having to worry about multiple counting, (this is possible because although the same property may be measured many times for the same patient, only one measurement value is considered to be valid at any given point in time). In the case of strict hierarchies, this feature is referred to as summarizability. Current data models do not specifically address this issue of correct aggregation in the case of non-strict hierarchies.

 Another requirement related to classification structures is that they should be able to handle temporal change. Classifications change and new diagnoses and new groups come and go at a steady rate. The CDW should support this in an intelligent way, so that analysis of data across changes is handled smoothly and preferably transparently to the user.

 Continuously Valued Data. Measurements and lab results are the key facts in the CDW. Unlike typical DW facts, these types of data clearly do not yield any meaning when summed. The CDW should be able to support these advanced operations very efficiently, to supply the performance necessary to analyze large amounts of data accumulated over long periods of time. To do so, it must be investigated how pre-stored and pre-aggregated data can be used to achieve high performance. 

 Dimensionally Reduced Data. In a clinical DW, average patients might have hundreds of different facts describing their current situation; in diabetes treatment, about 200 facts are recorded. There is an urgent need to be able to aggregate this massive amount of information in a useful way. These could be combined into one aggregate measure indicating the overall lifestyle of the patient. In a traditional OLAP world, the only way to reduce dimensionality is by projection, thereby ignoring all information about the omitted dimensions. The dimension reduction approach clearly has advantages over this, as the complexity of the data is reduced, while the essence is maintained. The clinical DW should be able to support the definition of such combination functions, and it should provide good performance for reducing/increasing the number of dimensions. 

 Integration of Very Complex Data. Very complex types of data, for example high-resolution medical images, also characterize the clinical world. It should be possible to incorporate this type of data in the CDW for data analysis purposes. The functionality should be more advanced than just allowing the raw data to be stored and retrieved. Rather, it should be possible to define feature extractors on the raw data, e.g., pattern recognition functions for wounds, and to perform analyses on the extracted features. The extractors should be tightly integrated with the DW, allowing for addition of new and modification of existing extractors incrementally, i.e., without having to recompute every feature from scratch. 

 Support for Clinical Protocols. The introduction of managed care is a very prominent current trend in the clinical world. Instead of relying solely on the judgment and knowledge of one doctor, the treatment of specific diseases is conducted according to well-defined protocols that specify the conditions and actions for using specific treatments. The protocol can be viewed as a “best practice" or an advanced set of business rules. A patient can be treated according to different protocols at different times. There is a need to analyze the actual treatments, to investigate conformance to the protocols, outcomes, etc. The queries against the CDW could be generated directly from the protocols, and the results of these be used to test conformance, to adjust the protocols, etc. The CDW should have integrated support for clinical protocols, to accommodate this important part of clinical practice. 

 Support for Medical Research. Medical research can take several different forms; one form that the clinical DW enables is the so-called qualitative research where large amounts of data is analyzed to confirm known or discover unknown trends and correlations in the data. The discovery process in medical research would benefit enormously from having data mining facilities integrated into the CDW. There should be a conceptually simple, fast performing, and yet flexible way to produce the “flat" sets of data that are normally fed into data mining algorithms. The results could be used as inspiration for hypotheses that could then be tested in controlled, formal clinical studies. The integration of data mining and data warehousing and the use of a DW for research purposes are both utilized in today’s DW products.

** On-Line Analytical Processing (OLAP) systems for medical applications**

*Using pre-aggregation in OLAP*

 On-Line Analytical Processing (OLAP) systems, which aim to ease the process of extracting useful information from large amounts of detailed transactional data, have gained widespread acceptance in traditional business applications as well as in new applications such as health care. These systems generally offer a dimensional view of data, in which measured values, termed facts, are characterized by descriptive values, drawn from a number of dimensions; and the values of a dimension are typically organized in a containment-type hierarchy. A prototypical query applies an aggregate function, such as average, to the facts characterized by specific values from the dimensions.

 Fast response times are required from these systems, even for queries that aggregate large amounts of data. The perhaps most central technique used for meeting this requirement is termed pre-aggregation, where the results of aggregate queries are pre-computed and stored, i.e., materialized, for later use during query processing. Pre-aggregation has attracted substantial attention in the research community, where it has been investigated how to optimally use pre-aggregated data for query optimization and how to maintain the pre-aggregated data when base data is updated. Further, the latest versions of commercial products offer query optimization based on pre-computed aggregates and automatic maintenance of the stored aggregates when base data is updated.

 The fastest response times may be achieved when materializing aggregate results corresponding to all combinations of dimension values across all dimensions, termed full pre-aggregation. However, the required storage space grows rapidly, to quickly become prohibitive, as the complexity of the application increases. This phenomenon is called data explosion and occurs because the number of possible aggregation combinations grows rapidly when the number of dimensions increase, while the sparseness of the multidimensional space decreases in higher dimension levels, meaning that aggregates at higher levels take up nearly as much space as lower-level aggregates. 

 The premise underlying the applicability of practical pre-aggregation is that lower-level aggregates can be re-used to compute higher-level aggregates, known as summarizability. Summarizability occurs when the relationships between facts and dimensions are many-to-one and facts are always mapped to the lowest levels in the dimensions. However, the data encountered in many real-world applications fail to comply with this rigid regime. The issue is to transform dimension hierarchies to obtain summarizability, and then to integrate the transformed hierarchies into current systems, transparently to the user, so that standard OLAP technology is re-used. The overall idea is to take un-normalized MOs and transform them into normalized MOs that are well supported by the practical pre-aggregation techniques available in current OLAP systems. Queries are then evaluated on the transformed MOs. However, we still want the users to see only the original MOs, as they reflect the users’ understanding of the domain. This prompts the need for means of handling both the original and the transformed MOs. 

**Fig. 2:**
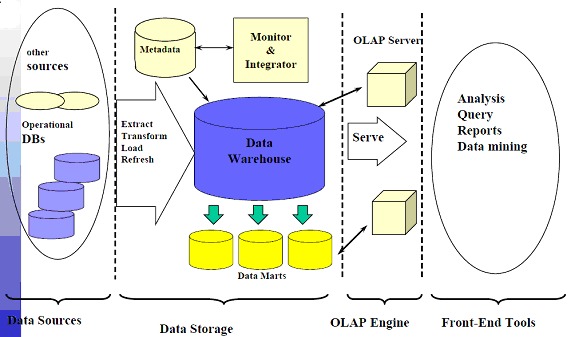
Architecture of DW and OLAP integration

 A current trend in commercial OLAP technology is the separation of the front-end presentation layer from the back-end database server. Modern OLAP applications consist of an OLAP client that handles the user interface and an OLAP server that manages the data and processes queries. The architecture of such a system is given to the left in Fig. 2. 

This separation of client and server facilitates our desire to have the user see the original MO while queries are evaluated against the transformed MO. The OLAP server for navigation data needs to support dimension hierarchies which have non-summarizable properties, a requirement not yet supported by many commercial systems today. However, relational OLAP systems are able to support this type of hierarchies. If the OLAP system available does not have sufficiently flexible hierarchy support, one solution is to build a special-purpose OLAP server that conforms to the given API. We note that the only data needed to answer navigational queries is the hierarchy definitions. Thus, we only need to store the fact data (facts and fact-dimension relations, in the proposed MMDM) once, in the aggregation data, meaning that the overall storage requirement is only slightly larger than storing just the aggregation data. Navigational queries are evaluated on the original hierarchy definitions and do not need to be re-written by the query handler.

*Extending OLAP Querying to Relational Object Databases*

 Specifically designed with the aim of better supporting the retrieval of higher-level summary information from detail data, OLAP systems offer substantial additional user-friendliness over general database management systems (DBMSs). The special dimensional data models employed in OLAP systems enable visual querying, as well as contribute to enable OLAP systems to offer better performance for aggregate queries than do traditional DBMSs. As another example, most OLAP systems support automatic aggregation, which means that the system knows which aggregate functions to apply when retrieving different higher-level summaries.

 Almost all OLAP systems are based on a dimensional view of data, in which measured values, termed facts, are characterized by descriptive values drawn from a number of dimensions; and the values of a dimension are typically organized in a containment-type hierarchy. While the dimensional view of data is particularly well suited for the aggregation queries performed in OLAP analysis, it also limits the abilities of OLAP systems to capture complex relationships in the data. As a result, an OLAP database only captures some of the structure available in the data from which it derives. Furthermore, it is often difficult or impossible to combine data from an OLAP system with data from other sources.

When integrating data from databases based on different data models, the traditional approach has been to map all data into one common data model and federate the (logically) transformed data rather than the original data. An alternative approach is to combine data from summary databases (SDBs) and object databases (ODBs), data being handled using the most appropriate data model and database technology: SDB systems for summary data and ODB systems for complex, general data. Many reasons exist for preferring federating SDBs with ODBs, as opposed to physically integrating these. The generic arguments for federation include leveraging existing technology, accessing the most current information, and allowing the autonomous existence of the systems being federated [**18**]. 

In many situations, SDBs only contain abstract summary data and do not contain the base data from which the summary data is derived, thus rendering access to external databases necessary to be able to answer certain queries. Federating SDBs and ODBs enables a simple and special-purpose SDB system. An SDB needs not contain all objects, attributes, and relationships in the base database, but only the elements relevant to summary querying. This is attractive, as capturing all information in the SDB unnecessarily impedes casual use of the SDB system. Indeed, most OLAP systems that implement summary databases do not have the necessary facilities, e.g., category inheritance, to support this extra information.

 The federated approach allows the SDB to contain only the most commonly used information, providing a simple summary-level view of data, while still allowing access to relevant data that resides in the SDB. When SDB data resides in a special-purpose SDB system, we cannot use existing database middleware to access it, leading to a need for technology that enables federations of SDBs and ODBs.

 It is possible to obtain better performance when performing summary querying in an OLAP-type system rather than in a general-purpose DBMS. The former type of system typically employs specialized, performance enhancing techniques, such as multidimensional storage and pre-aggregation. Therefore, even if all data comes from one single (non-SDB) database, it is desirable to perform summary querying in a specialized OLAP system. Next, it is easier to formulate summary queries in an SDB system than in a general (relational or object) DBMS. This is because an SDB query language is designed exclusively for expressing summary queries over categories, taking advantage of, e.g., the automatic aggregation implied by the summary database semantics. Even when extending an SDB language to access object data, it is easier to pose summary queries in the extended language than in a general database query language such as SQL.

 An SDB system may support the formulation of summary queries that return correct, or meaningful, query results. When building an SDB, the data may be shaped in order to satisfy summarizability conditions. For example, if patients have several diseases, and we summarize over all diseases to get the total number of sick people, we will get the wrong result as some patients are counted more than once. We may enrich an SDB system with information that enables the system to ensure correctness. The federated approach offers additional flexibility when the query requirements change. SDBs may be huge, and therefore rebuilding them may be time consuming.

 Updates to an SDB, e.g., adding new types of information, may require a total or partial rebuild of the database. Because of the rebuild time, a rebuild of the SDB will most likely be refused or postponed to the next scheduled rebuild, e.g., once a week or once a month. In contrast, a new link can be added in a matter of minutes, yielding much faster access to newly required information. This allows rapid prototyping of OLAP systems. In a relational DB setting, the ability to do this rapid prototyping is one of the key selling points.

## Conclusions

 Motivated by the increasingly widespread use of OLAP technology, we have presented the concepts and techniques underlying a system that logically integrates data in OLAP databases with data from outside object databases, without requiring physical integration of the data.

 Summary data is best handled using OLAP technology, while complex detail level data structures are best handled with object database technology. This enables the handling of the data using the most appropriate data model and technology, while still allowing queries to reference data across the different databases and data models. 

 An extended multidimensional data model, which could be implemented by using standard OLAP technology and techniques such as pre-aggregation, was presented. The scope of practical pre-aggregation was significantly extended to cover a much wider range of realistic situations.

 Finally, the paper presents the concepts and techniques underlying a flexible federated system for extending OLAP querying to external object databases. The system eased the integration of OLAP data with complex external data considerably and allowed data to be handled using the most appropriate data model and technology: OLAP systems for dimensional data and object database systems for more complex, general data.
